# Early Detection of Pancreatic Cancer: Role of Biomarkers in Pancreatic Fluid Samples

**DOI:** 10.3390/diagnostics10121056

**Published:** 2020-12-06

**Authors:** Noboru Ideno, Yasuhisa Mori, Masafumi Nakamura, Takao Ohtsuka

**Affiliations:** 1 Department of Surgery and Oncology, Graduate School of Medical Sciences, Kyushu University, Fukuoka 812-8582, Japan; ideno@surg1.med.kyushu-u.ac.jp (N.I.); y-mori@surg1.med.kyushu-u.ac.jp (Y.M.); mnaka@surg1.med.kyushu-u.ac.jp (M.N.); 2 Department of Digestive Surgery, Breast and Thyroid Surgery, Graduate School of Medical Sciences, Kagoshima University, Kagoshima 890-8520, Japan

**Keywords:** pancreatic cancer, pancreatic juice, duodenal juice/fluid

## Abstract

Pancreatic ductal adenocarcinoma (PDAC) is the fourth leading cause of cancer-related deaths worldwide. Most patients with PDAC present with symptomatic, surgically unresectable disease. Therefore, the establishment of strategies for the early detection is urgently needed. Molecular biomarkers might be useful in various phases of a strategy to identify high-risk individuals in the general population and to detect high-risk lesions during intense surveillance programs combined with imaging modalities. However, the low sensitivity and specificity of biomarkers currently available for PDAC, such as carbohydrate 19-9 (CA19-9), contribute to the late diagnosis of this deadly disease. Although almost all classes of biomarker assays have been studied, most of them are used in the context of symptomatic diseases. Compared to other body fluids, pancreatic juice and duodenal fluid are better sources of DNA, RNA, proteins, and exosomes derived from neoplastic cells and have the potential to increase the sensitivity/specificity of these biomarkers. The number of studies using duodenal fluid with or without secretin stimulation for DNA/protein marker tests have been increasing because of the less-invasiveness in comparison to pancreatic juice collection by endoscopic retrograde cholangiopancreatography (ERCP) and endoscopic ultrasound-guided fine needle aspiration (EUS-FNA). Genomic analyses have been very well-studied, and based on PDAC progression model, mutations detected in pancreatic juice/duodenal fluid seem to indicate the presence of microscopic precursors and high-grade dysplasia/invasive cancer. In addition to known proteins overexpressed both in precursors and PDACs, such as CEA and S100P, comprehensive proteomic analysis of pancreatic juice from patients with PDAC identified many proteins which were not previously described. A novel technique to isolate exosomes from pancreatic juice was recently invented and identification of exosomal microRNA’s 21 and 155 could be biomarkers for diagnosis of PDAC. Since many studies have explored biomarkers in fluid samples containing pancreatic juice and reported excellent diagnostic accuracy, we need to discuss how these biomarker assays can be validated and utilized in the strategy of early detection of PDAC.

## 1. Introduction

In contrast to the decline in cancer-related deaths from other malignancies, progress in the managing pancreatic ductal adenocarcinoma (PDAC) has been slow, and the incidence of cancer-related deaths due to PDAC continues to rise. The 5-year relative survival rate is as low as 8% for all stages [1]. Despite of these grim numbers, there is unequivocal evidence that diagnosis of PDAC at earlier, resectable stages has a profoundly favorable impact on the prognosis. The 5-year survival rate of resected PDAC is as high as ~25–30% in major treatment centers, increasing to 30–60% for tumors <2 cm, and as high as 75% for “minute” lesions under 10 mm in size [2]. Thus, early detection of PDAC is an area of utmost priority. The current diagnosis of pancreatic cancer relies on a combination of medical history and physical examination, serum tumor markers such as carbohydrate 19-9 (CA19-9) and carcinoembryonic antigen (CEA), imaging studies (computed tomography (CT), magnetic resonance imaging (MRI), endoscopic ultrasound (EUS)), and pathological confirmation with a tissue or fluid samples from EUS-fine-needle aspiration (FNA) or pancreatic juice (PJ). However, none of these strategies alone or in combination provide satisfactory sensitivity and specificity for the early diagnosis of pancreatic cancer, which is unusual and typically incidental, since pancreatic cancer patients seldom exhibit disease-specific symptoms until late in the course of the disease [2]. Although the goal of early detection of PDAC is laudable and likely to result in significant improvement in overall survival, the relatively low incidence of PDAC makes general population screening infeasible [1]. Thus, the following two strategies should be established for the early detection of PDACs. One is the identification of high-risk individuals in the general population who need a longitudinal surveillance program. Recently, various subgroups at higher risk for PDAC have been identified, including those with familial risk due to germline mutations, a history of pancreatitis, patients with mucinous pancreatic cysts, and elderly patients with new-onset diabetes [3]. Another is the development of appropriate diagnostic/surveillance biomarkers and imaging-based modalities. Many published studies to date have explored minimally invasive or non-invasive biomarkers in blood, urine, stool, saliva, or PJ for screening or early detection of PDAC. Compared to the other biological fluids, PJ has multiple advantages. It reflects the function of the whole pancreas, and, most importantly, it contains the highest concentration of proteins, DNA and exosomes released from the normal or injured pancreas and potentially increases the sensitivity to detect biomarkers derived from PDAC and the high-risk lesions, including pancreatic intraepithelial neoplasia (PanIN) and mucinous pancreatic cysts [4]. This review provides an overview of current insights into studies focusing on screening and early detection of pancreatic cancer using PJ/duodenal fluid (DF) samples and discusses how tests can be conducted in the actual clinical settings.

## 2. Methods of Collecting PJ/DF Samples

**Figure 1:**
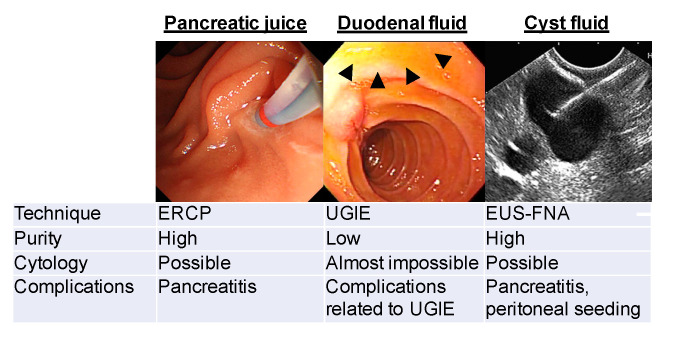
Representative methods to collect pancreatic juice. ERCP: endoscopic retrograde cholangiopancreatography; GIE: gastrointestinal endoscopy; EUS-ENA: endoscopic ultrasonography-guided fine needle aspiration; PJ: pancreatic juice. Black triangle: accumulated duodenal fluid. Scale bar: 1 cm.

There are currently 3 methods of collecting PJ or DF using an endoscope, as demonstrated in Figure 1.

In 1974, Cotton et al. [5] collected pure PJ under direct visualization using a catheter placed directly in the main pancreatic duct during endoscopic retrograde cholangiopancreatography (ERCP). This is the purest PJ from the entire pancreatic ductal system. Unfortunately, 1–25% of patients experienced post-ERCP pancreatitis [6,7,8], and routine ERCP for sampling is not currently recommended.

In 1999, a less invasive technique to obtain PJ was invented by Raimondo et al. [9]. During routine upper endoscopy and after intravenous secretin administration, an aspiration catheter was passed into the duodenum through the endoscope channel, where DF was collected without cannulation of the papilla. In 2003, Conwell et al. [10] reported a similar method for PJ collection by direct aspiration of DF through the suction channel of the endoscope without using a catheter.

In our previous study, we investigated whether sufficient amount of DF could be collected at the time of ERCP without secretin administration. After positioning the side-view endoscope in the second portion of the duodenum, DF was collected for 5 min by gently pushing the duodenal mucosa using an ERCP cannula that had 2 side holes near the tip with another end hole. Thereafter, secretin (1 μg/body) was administered intravenously, and the DF was continuously collected for an additional 10 min. The average volume during the initial 5 min without secretin administration was 2.3 ± 1.8 mL. Although the volumes from 0 to 5 min and 5 to 10 min after secretin administration were significantly larger than those without secretin administration, we concluded that secretin administration was not necessary to measure protein concentration [11] and detect mutated genes in neoplastic cells [12]. A multi-institutional validation study reported that a sufficient amount of DF was collected when screening upper gastrointestinal endoscopy using a forward-view endoscope with an ERCP cannula without secretin administration [13].

Suenaga et al. [14] described a new method of collecting secretin-stimulated PJ to minimize contamination of other gastrointestinal fluids such as gastric juice. They used a disposable endoscopic cap placed over the major papilla to collect secretin-stimulated PJ from the ampulla. Importantly, the median total mutation concentration of PJ in samples collected with the cap method was higher than in those collected without the cap.

Although the current Japanese consensus guidelines do not recommend endoscopic ultrasound-guided fine needle aspiration (EUS-FNA) in cases of suspected mucinous cystic neoplasm, the American Society for Gastrointestinal Endoscopy Guideline attached the importance of EUS-FNA for cytology, measurements of pancreatic enzymes and tumor markers for the differential diagnosis and tumor grading [15,16,17]. Complications to be considered in EUS-FNA are pancreatitis and peritoneal seeding when cysts contain malignant cells.

## 3. Genomic Analysis of PJ/DF for Early Detection of PDAC

Genetic alterations in PDAC have been well characterized. It is clear that specific cancer-associated genes are targeted in PDAC and that multiple genes are often mutated in a single cancer. Four genes were recurrently altered, including KRAS, TP53, CDKN2A, and SMAD4, with frequencies >90%, 50–75%, >95%, and 55%, respectively [18]. Mutations of KRAS are considered to be “founder mutation,” because more than 90% of precursor lesions of PDAC, low-grade PanINs, harbor activating mutations of the gene [19]. The frequencies of the other mutations seem to be increased according to the PanIN progression [20], which indicates that progressor mutations are possibly useful markers to distinguish early invasive cancer and high-grade from low-grade dysplasia accurately.

### 3.1. Identification of Microscopic Precursor Lesions Using DF Samples

Pancreatic imaging tests can identify patients with pancreatic neoplastic cysts but not microscopic dysplasia. Few studies have investigated genetic alterations in PJ or DF from asymptomatic patients. Sadakari et al. [21] performed KRAS mutational analysis using secretin-stimulated PJ/DF collected from the pancreatic duct and the duodenal lumens of 36 patients with subtle pancreatic parenchymal abnormalities by EUS in a series of Cancer of the Pancreas Screening (CAPS) study. These asymptomatic subjects had a family history of pancreatic cancer or inherited predisposition to pancreatic cancer. Even though only 7 had small pancreatic cysts, and none had pancreatic cancer, KRAS mutation was detected in 29 of 36 (81%) pancreatic duct fluid samples. Of these 29 patients, 23 (79.3%) had mutations detected in their DF. In 2015, Eshleman et al. [22] also reported a high frequency of KRAS mutations (96/194, 50%) in the DF samples from other asymptomatic patients undergoing pancreatic screening as part of the CAPS studies. They have maintained long-term surveillance of these patients, and 6 patients who required pancreatectomy were diagnosed as having low-grade~high-grade PanINs or intraductal papillary mucinous neoplasms (IPMNs), which indicates that mutant KRAS detected in pancreatic fluid samples may provide evidence that the pancreas contains PanINs.

### 3.2. Screening for High-Grade Dysplasia and Invasive Cancer

Detection of alterations of TP53, CDKN2A, and SMAD4 in the DF were sought to identify high-grade dysplasia and early-stage invasive PDAC in asymptomatic individuals. In 2013, Kanda et al. [23] confirmed the prevalence of TP53 mutations in precursors and PDAC using resected specimens. Mutations were detected in 5.4% of low-grade PanIN/IPMN, 42.8% of high-grade PanIN/IPMN, and 75% of PDAC. Consistent with the prevalence detected in tissue samples, TP53 mutations were detected by digital high-resolution melting analysis (HRM) in secretin-stimulated DF samples of 7.1% of low-grade PanIN/IPMN, 50% of high-grade PanIN/IPMN, and 67.4% of PDAC. In 2017, Yu et al. [24] developed a novel next-generation sequencing method to detect low concentrations (0.1% to 1%) of mutations in DF. In addition to the sensitive detection of mutations in TP53 and/or SMAD4 (22 of 34 cases with PDAC, 64.7%), they found that DF concentrations, particularly mutations affecting TP53 and SMAD4, could distinguish PDAC from IPMN cases with 32.4% sensitivity and 100% specificity (area under the curve, AUC 0.73, *p* = 0.00002) and controls (AUC 0.82, *p* < 0.0001).

In 2018, Singhi et al. [25] reported highly accurate preoperative cyst fluid analyses to detect advanced pancreatic cystic neoplasms. They assessed 626 pancreatic cystic fluid via EUS-FNA using targeted next-generation sequencing (NGS). Based on analyses of 102 matched surgically removed specimens, they found that the combination of KRAS/GNAS mutations and alterations in TP53/PIK3CA/PTEN had 89 sensitivity and 100% specificity for high-grade IPMN and invasive IPMN.

### 3.3. Differentiation of Cystic Lesions of the Pancreas

IPMN and MCN are cystic precursor lesions of pancreatic cancer. Although pancreatic imaging tests such as EUS and MRCP can detect cystic lesions including these neoplasms, these tests do not reliably predict its pathology. MCN will be easier to characterize because of its particular radiographic image findings and the etiology. On the other hand, it is sometimes difficult in IPMN if communication between cysts and the pancreatic duct is not evident in these imaging tests. In 2011, Wu et al. [26] identified GNAS mutations as IPMN-specific genetic alterations. In 2013, Kanda et al. [27] reported GNAS mutations were detected in secretin-stimulated DF of 50 of 78 familial and sporadic cases of IPMNs (64.1%), 15 of 33 (45.5%) with only small cysts (<5 mm), but none of 57 disease controls. Considering that the frequency of GNAS mutations is 60–70% in IPMNs, DNA test by DF, not by cyst fluid from EUS-FNA, seems to be sufficient to differentiate IPMN from the other benign cyst. To make this DNA test less invasive, we reported GNAS mutations in DF without secretin stimulation in patients with IPMN using real-time PCR assay with TagMan probe [12].

### 3.4. Biomarker Proteins in PJ/DF from Patients with PDAC

PJ contains cancer-specific proteins rendering it a promising tool for identifying novel biomarkers in pancreatic ductal adenocarcinoma. Earlier studies reported the detections of mRNAs/proteins in PJ, which are expressed in pancreatic cancer tissues. Recent comprehensive proteomics technologies have been applied to PJ to identify unknown biomarkers in PDAC.

Carcinoembryonic antigen cell adhesion molecule 5 (CEACAM5, CEA) is a cell surface protein which is used as a clinical biomarker for gastrointestinal cancers and may promote tumor development through its role as a cell adhesion molecule. CEA is one of the representative molecules that has been investigated in many studies for the diagnosis of pancreatic neoplasms using PJ [28,29,30,31,32,33,34].

CEA levels were essentially higher in pure PJ from pancreatic cancer patients than in those from chronic pancreatitis patients or controls with the normal pancreas and significant diagnostic advantage to sera [30]. Comprehensive proteomic analysis of PJ also identified CEA as a protein overexpressed in pancreatic cancer [31]. Based on these results, we and others tested the diagnostic ability of CEA in DF. In the initial study, we performed with a small number of control subjects, and the concentrations of CEA in DF obtained from patients with PDAC were significantly higher than those in the control group [11]. However, in our validation study, including 94 PDAC patients and 61 control subjects with normal pancreas, the significant difference was disappeared [13], consistent with another study [28].

CEA in cyst fluid from mucinous pancreatic cysts seems to be a useful biomarker to differentiate from non-neoplastic cysts and predicts malignancy in combination with the other parameters. In 2004, Brugge et al. [33] reported that the measurement of CEA levels in cyst fluid obtained by EUS-FNA was an accurate test useful for the differential diagnosis of mucinous cystic neoplasms from nonmucinous cystic lesions. Kawai et al. [34] also reported that malignant IPMNs displayed significantly higher CEA levels in PJ compared to benign IPMNs. In 2009, Maire et al. [35] reported that the negative predictive value of CEA and CA72.4 in pancreatic cyst fluid was 96% and 96%, respectively, for the preoperative differential diagnosis of benign versus malignant IPMN. In 2012, Hirono et al. [32] identified CEA levels of more than 30 ng/mL in PJ obtained from preoperative ERP and mural nodule size of more than 5 mm in branch duct IPMN as independent predictive factors of malignant BD-IPMN.

Biomarkers for the detection of earlier-stage PDAC should be associated with early-phase pancreatic carcinogenesis. S100 family proteins are small Ca2+-binding EF-hand-type proteins that affect the regulation of several intracellular and extracellular processes, including cell proliferation, differentiation, and intracellular signaling. In 2006, Ohuchida et al. [36] reported S100 calcium-binding protein P (S100P) expressed in PDAC, PanIN, and IPMN, but not in normal pancreatic ductal cells, indicating that S100P is an early developmental marker for PDAC. Importantly, S100P is a secreted protein that is measurable in PJ. Based on this study, we tested the diagnostic ability of S100P in the DF. As expected, S100P concentrations were significantly higher in duodenal fluid from patients with PDAC than in healthy controls. A logistic regression model that included age showed that the sensitivity and specificity of S100P concentration in diagnosing stage 0/IA/IB/IIA PDAC was 85% and 77%, respectively, with an area under the receiver operating characteristic curve of 0.82. These results indicate that the measurement of S100P in the DF may serve as a useful screening test for the detection of PDAC [11,13].

Comprehensive proteomic analysis of PJ from patients with PDAC identified many proteins, including known pancreatic cancer tumor markers and proteins overexpressed in pancreatic cancers. These studies also demonstrated potential biomarkers that have not been previously described in PJ from pancreatic cancer patients, as listed in Table 1 [31,37,38]. These data highlight the potential value of biomarkers from various biological sources in the early diagnosis of pancreatic cancer. However, in proteomic analyses, factors that are normally involved at the time of PJ collection, such as main pancreatic duct obstruction and contamination of blood and bile, strongly affected the protein composition of PJ [39]. Therefore, newly identified biomarkers must be validated in a larger patient population.

### 3.5. Analysis of miRNA in Pancreatic Juice

MicroRNAs (miRNAs) have emerged as a new class of biomarkers that exhibit various oncogenic and tumor suppressor functions thorough messenger RNA (mRNA) silencing and post-transcriptional gene regulation. In addition to other malignancies, several miRNAs, including miR-21, miR-196a-2, miR-155, and miR-210 were differentially expressed in pancreatic cancer tissues or cell lines [40,41,42,43]. In 2010, Sadakari et al. [44] first reported that higher expression of miR-21 and miR-155 in PJ was a potential diagnostic marker for PDACs that did not detect atypical cells in preoperative pancreatic cytology.

Exosomes are 40–150 nm lipid bilayer membrane-bound particles derived from specific biogenesis pathways within cells and are accessible within the plasma of the circulating peripheral blood [45]. In 2019, Nakamura et al. [46] reported successful extraction of exosomes from PJ in patients with PDAC. The exosomes contained microRNAs (ex-miRs), which are supposed to be protected from endogenous RNase activity [47]. Based on our previous work [44], expression of Ex-miR-21 and Ex-miR-155 from PJ was compared between patients with PDAC and those with chronic pancreatitis. Either ex-miRs from 27 PDAC patients were significantly higher than those from 8 chronic pancreatitis patients. In addition, when combining the results of ex-miR-21 with ex-miR-155, the sensitivity, specificity, and accuracy of PDAC diagnosis were 96%, 75%, and 91%, respectively, which were better than those of PJ cytology.

## 4. Conclusions

**Figure 2:**
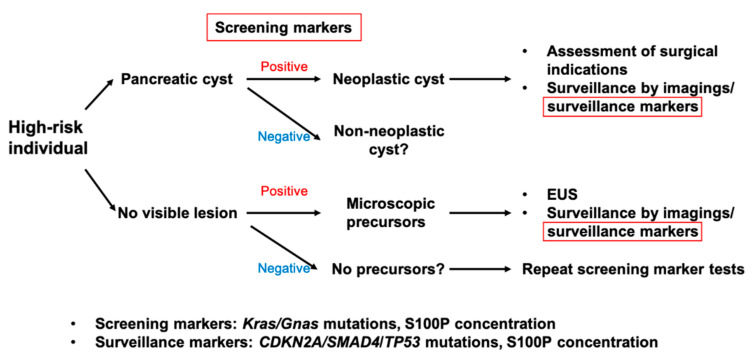
A proposed strategy for early detection of pancreatic cancer and the role of screening/surveillance biomarkers tested by fluid samples obtained by an endoscope. DF will be recommended as fluid sample.

The PJ has the potential to provide evidence of the presence or absence of dysplasia and cancer, which are not evident on imaging. Pure PJ obtained via ERP would be an ideal source for biomarker detection. However, purity seems to be becoming less important because DF contains sufficient cells or proteins derived from pancreatic tumors to detect genetic alterations or novel protein markers. Based on our current understanding of the relationships between biological process and natural history of pancreatic cancer, each biomarker plays a role in the early detection of PDAC, as presented in Figure 2. For example, *KRAS* mutations, more than 90% of low-grade PanINs harbor the mutations of this, would be representative screening biomarkers in individuals at high risk of pancreatic cysts or apparently normal pancreas. In the longitudinal surveillance of patients with mucinous pancreatic cysts or those who are suspected of having microscopic precursors by screening tests, surveillance markers with high sensitivity and specificity would be used in combination with imaging modalities. In addition to identifying genetic alterations observed in the late phase of PDAC carcinogenesis, increasing the concentration of screening biomarkers might equally be useful. Based on our previous works [11,13], a multi-institutional study to validate S100P test in DF from PDAC patients is ongoing. Further studies, including ours, will provide accurate biomarker assays with minimal invasion, which satisfy strategies for early detection of PDAC in asymptomatic individuals.

## Figures and Tables

**Table 1:** Comprehensive proteomic analyses of pancreatic juice from PDAC patients.

Authors (Ref)	Year	Control Cohort	Number of Unique Proteins in PJ from PDAC	Identified Protein Previously Undescribed in PDAC
Grønborg et al. [31]	2004	N/A	170	pg96, Azurocidin
Chen et al. [37]	2007	Chronic pancreatitis	21	Plasminogen, NCAM L1, Caldecrin
Tian et al. [38]	2008	Cancer-free	24	DJ-1, AIBG

PDAC: pancreatic ductal adenocarcinoma; PJ: pancreatic juice.
